# Perirenal schwannoma: a case report

**DOI:** 10.1186/1752-1947-2-189

**Published:** 2008-06-02

**Authors:** Mona El-Bahrawy, Bijan Khoubehi, David Hrouda

**Affiliations:** 1 Department of Histopathology, Division of Investigative Science, Imperial College, Hammersmith Hospital, DuCane Road, London, W12 0NN, UK; 2 Department of Urology, Charing Cross Hospital, Fulham Palace Road, London, W6 9NT, UK

## Abstract

**Introduction:**

Nerve sheath tumours of the kidney are particularly rare and, in the few reported cases, are all situated in the hilar region.

**Case presentation:**

We describe the case of a tumour presenting towards the lateral border of the ventral aspect of the mid-zone of the kidney. This was a spindle cell lesion in which the cells strongly and diffusely expressed cytokeratins, but were negative for epithelial membrane antigen. The cells also expressed S-100 protein and glial fibrillary acidic protein, confirming the diagnosis of a cellular schwannoma.

**Conclusion:**

To the best of our knowledge, this is the first case of a cellular schwannoma presenting towards the lateral border of the kidney. The case also highlights the importance of using a panel of antibodies in diagnosing spindle cell neoplasms in the kidney.

## Introduction

Schwannomas are encapsulated nerve sheath tumours that occur at all ages, but are most common between the ages of 20 and 50 years and affect both genders equally. The tumours have a predilection for the head, neck and flexor surfaces of the upper and lower extremities. Deeply situated tumours predominate in the posterior mediastinum and the retroperitoneum [1]. A schwannoma is a slowly growing tumour that is usually present several years before diagnosis. Schwannomas behave in a benign fashion and malignant change is rare [2,3].

## Case presentation

A 55-year-old man presented with lower urinary tract symptoms in the form of hesitancy, poor stream and urgency. The patient did not have any flank and/or colicky pain. He had no history of urinary tract-related diseases or previous related illnesses and was constitutionally well with good appetite and normal body mass index. No abnormalities were detected on clinical examination.

Laboratory investigations revealed that serum prostate specific antigen was 5.1 ng/ml and serum creatinine was 86 μmol/l. Urine microscopy revealed no atypical cells and no haematuria. Prostate core biopsies showed benign prostatic hyperplasia.

**Figure 1:**
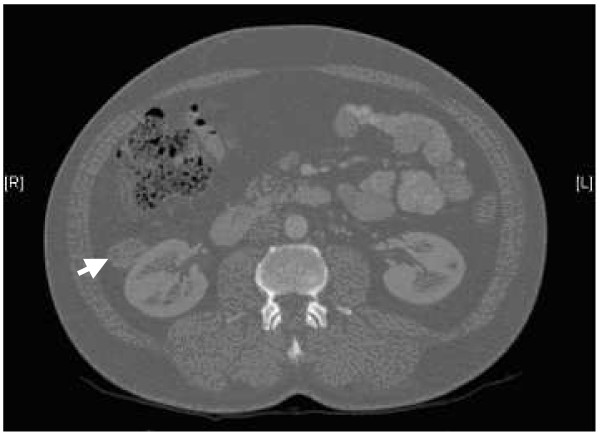
A computed tomography scan showing a right renal mass present on the ventral aspect of the kidney away from the hilar region (arrow).

An incidental exophytic lesion measuring 3 × 3.2 × 4.2 cm was discovered in the right kidney on abdominal ultrasound. This was a homogeneous hypoechoic structure with a well-defined margin seen lying towards the lateral border of the ventral aspect of the mid-zone of the kidney. The lesion appeared as a soft tissue abnormality on contrast renal computed tomography (Figure 1). There was a small area of calcification within it. The maximum diameter was approximately 2.17 cm.

The lesion was removed by laparoscopic partial nephrectomy with a small amount of renal parenchyma and surrounding fat with clear surgical margins as a curative approach. The specimen comprised a nodule surrounded by fat and attached with a small pedicle to a piece of renal tissue. The nodule was well-circumscribed, seemingly encapsulated, firm in consistency and measured 2.5 × 1.4 × 2.5 cm. The cut surface was grey-white with microcystic areas.

**Figure 2:**
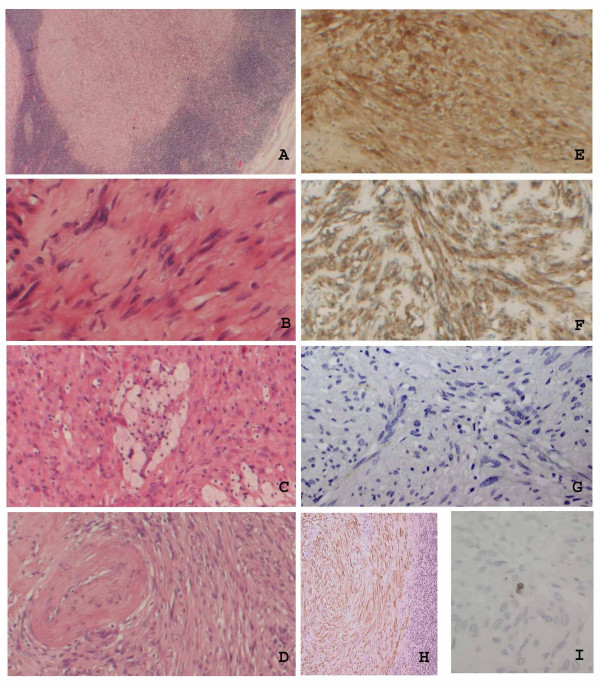
**Histological features of a schwannoma**. This is a spindle cell lesion surrounded by a rim of lymphocytes (A), ×100. The lesion is composed of fascicles of bland spindle cells (B), magnification ×600. There are aggregates of foamy histiocytes (C), magnification ×400. Thick-walled blood vessels are focally present (D), magnification ×200. The cells express S-100 protein (E), glial fibrillary acidic protein (F), magnification ×100; are negative for CK7 (G), magnification ×200; but express broad range cytokeratins (H), magnification ×100. The Ki67 index is very low, less than 1% (I), magnification ×400.

Microscopic examination showed a well-circumscribed, partially encapsulated spindle cell lesion. The spindle cells were arranged in whorls and intersecting fascicles with focal intervening sclerosis. The tumour displayed relatively uniform cellularity. No cytological atypia, necrosis or mitoses were present. Thick-walled, hyalinised blood vessels and aggregates of foamy macrophages were present (Figure 2A–D). A cuff of lymphoid tissue surrounded the tumour.

Immunostaining for a panel of cytokeratins was performed including broad range cytokeratins (AE1/AE3) and CK 7. The cells strongly and diffusely expressed cytokeratins on using a broad range cytokeratin cocktail, but were negative for CK7. The cells were also negative for epithelial membrane antigen (EMA). The tumour cells expressed S-100 protein and glial fibrillary acidic protein (GFAP) and were negative for HMB45, melan A, smooth muscle actin (SMA), desmin, CD34 and CD117. Immunostaining for Ki67 showed a very low index of less than 1% (Figure 2E–I).

The morphology and immunoprofile were characteristic of a cellular schwannoma.

## Discussion

A schwannoma is a benign nerve sheath tumour consisting of a highly ordered cellular component (Antoni A areas) and a loose myxoid component (Antoni B areas). Antoni A areas are composed of compact spindle cells that usually have wavy nuclei and indistinct cytoplasmic borders. The cells are arranged in interlacing fascicles and short bundles. In highly differentiated Antoni A areas, there may be nuclear palisading, whorling of the cells and Verocay bodies, formed by two compact rows of well-aligned nuclei separated by fibrillary cell processes. In Antoni B areas, the cells are arranged haphazardly in a loose matrix.

Cellular schwannoma is a rare variant of schwannomas, composed almost entirely of Antoni A areas that lack Verocay bodies. Small amounts of Antoni B may be present, usually not exceeding 10% of the lesion [4]. Underneath their capsule they may contain lymphoid aggregates. Cellular schwannoma occurs in a similar age group as classic schwannoma but tends to develop more often in deep structures, such as the posterior mediastinum and retroperitoneum. Initial scepticism was expressed about the biological behaviour of cellular schwannomas, with some suggesting that it was in fact a low-grade malignant peripheral nerve sheath tumour (MPNST). Woodruff et al. [5] stressed the benign clinical course of cellular schwannomas and the importance of distinguishing them from MPNST. Subsequent studies have confirmed the benign nature of this tumour [6,7].

Nerve sheath tumours of the kidney seem to be particularly rare, with only eight cases reported in the literature [8-10]. The renal hilum is a common site of renal schwannoma [11-13].

We have reported the case of a renal schwannoma. To the best of the authors' knowledge, this is the first case of a schwannoma reported towards the lateral border of the kidney rather than in the hilar region. On microscopic examination, this was a cellular schwannoma and hence did not show the typical features of a classical schwannoma, including the Antoni A and Antoni B areas. On immunostaining, the cells showed diffuse and strong positivity for cytokeratins. Focal cytokeratin expression has been reported in schwannomas [14], but in this case, the cytokeratin expression using a broad range cytokeratin cocktail was strong and seen in all cells. However, the cells were all negative for cytokeratin 7 and EMA. The strong cytokeratin expression with the atypical histological features could have led to a diagnosis of an epithelial renal neoplasm as a sarcomatoid carcinoma.

S-100 protein has been shown to have a broad distribution in human tissues, including the renal tubules. Lin et al. [15] studied the expression of S-100 protein in primary and metastatic renal cell carcinoma (RCC) and non-neoplastic renal tissue. The results demonstrated the nuclear and cytoplasmic staining pattern for S-100 protein in 56 (69%) out of 81 conventional (clear cell) RCCs, 10 (30%) out of 33 papillary RCCs, 1 (6%) out of 16 chromophobe RCCs, and 13 (87%) out of 15 oncocytomas. Focal immunostaining was present in 22 (92%) out of 24 normal renal tubules. A similar staining pattern was observed in 21 (70%) out of 30 metastatic RCCs. Importantly, 14.8% (12 out of 81) of clear cell RCC and 13.3% (4 out of 30) of metastatic RCC revealed an immunostaining profile of pancytokeratin (-)/S-100 protein (+). These data indicate that caution should be taken in interpreting an unknown primary with S-100 positivity and cytokeratin negativity [15]. This highlights the importance of using a panel of antibodies on handling a spindle cell lesion of the kidney to cover all of the possible differential diagnoses such as sarcomatoid carcinoma and synovial sarcoma in the context of the gross and histological features.

Different markers were used to exclude other spindle cell lesions. The markers for smooth muscle differentiation, desmin and SMA, were negative. Melan A and HMB45 were negative excluding a melanocytic lesion or an angiomyolipoma. CD34 and CD117 were used to exclude a gastrointestinal stromal tumour, whereas GFAP expression confirmed neural differentiation.

The patient presented with lower urinary tract symptoms, which were caused by benign prostatic hyperplasia, and the symptoms were not related to the renal neoplasm. The renal tumour was an incidental finding during routine investigations. There were no symptoms attributable to the lesion, which was completely asymptomatic. No flank pain and/or colic was ever experienced by the patient, and urine analysis did not show haematuria or atypical cells. The patient was constitutionally well, with a good appetite and normal body mass index and did not report any recent weight loss.

The differential diagnosis included RCC, therefore a curative approach was taken with laparoscopic partial nephrectomy including renal parenchyma with clear surgical margins.

A dimercaptosuccinic acid renal scan follow-up showed a differential renal function of 49% in the right kidney. Three years after the removal of the tumour the patient is well.

To the best of our knowledge, this is the first case of a renal schwannoma presenting towards the lateral border of the kidney. This was a cellular schwannoma, which showed, in addition to the expression of S-100 protein and GFAP, strong and diffuse expression of cytokeratins.

## Conclusion

Renal schwannomas, although rare, must be considered in the differential diagnosis of spindle cell lesions of the kidney. We report a case of an incidentally identified perirenal schwannoma, which was treated by partial nephrectomy. In 1988, Somers et al. [9] reported a case of renal schwannoma recommending radical nephrectomy as the treatment of choice as then very few cases had been reported and the natural history and potential for malignancy was uncertain. Now given the benign clinical course of schwannomas, nephron-sparing surgery seems to be appropriate if technically feasible and the diagnosis is considered pre-operatively.

## Abbreviations

EMA: epithelial membrane antigen; GFAP: glial fibrillary acidic protein; MPNST: malignant peripheral nerve sheath tumour; RCC: renal cell carcinoma; SMA: smooth muscle actin.

## Competing interests

The authors declare that they have no competing interests.

## Authors' contributions

MEB carried out the histopathological diagnosis and verification by immunohistochemistry, and drafted the manuscript. BK participated in the management of the patient and in writing the manuscript. DH performed the surgical procedure and revised the manuscript. All authors read and approved the final manuscript.

## Consent

Written informed consent was obtained from the patient for publication of this case report and any accompanying images. A copy of the written consent is available for review by the Editor-in-Chief of this journal.
